# S92. DISTINCT RISK FACTORS FOR OBSESSIVE AND COMPULSIVE SYMPTOMS IN SCHIZOPHRENIA

**DOI:** 10.1093/schbul/sby018.879

**Published:** 2018-04-01

**Authors:** Emilio Fernandez-Egea, Yulia Worbe, Miguel Bernardo, Trevor W Robbins

**Affiliations:** 1 University of Cambridge; 2 Saint-Antoine Hospital, Paris; 3 Barcelona Clinic Schizophrenia Unit. Hospital Clinic Barcelona

## Abstract

**Background:**

Obsessive-compulsive disorder (OCD) is common in schizophrenia patients treated with antipsy-chotics with significant anti-serotoninergic action. Clozapine was the first drug reported and is still the medication more frequently associated with OCD, along with olanzapine. The aim of this study was to study the OCD prevalence, clinical profile and associated severity factors, using electronic records of a large cohort of clozapine-medicated schizophrenic patients. Patients were routinely screened for OCD using standardised scales as well as relevant clinical, psychometric and demographic data.

**Methods:**

The electronic records of a large cohort of clozapine-medicated schizophrenia patients routinely screened for OCD using standard measures were used. The Obsessive Compulsive Inventory Revised version (OCI-R) was available from 118 cases and a 21 points cut-off threshold for OCD was defined.

**Results:**

OCD prevalence was 47% and significantly higher in patients on several medications including clozapine than on clozapine monotherapy (64% vs 31%; p=.001). Two OCI-R factors had significantly higher scores in these patients and were associated with distinct risk factors: checking behaviour (mean=5.1; SD=3.6), which correlated with length on clozapine treatment (r=.21; p=.026), and obsessing factor (mean=4.8; SD=3.6), which correlated with psychosis severity (r=.59; p=.001). However, these factors along with total OCI-R, did not correlate with either clozapine dose or plasma levels, after correcting for psychosis severity.

**Discussion:**

We propose an imbalance between impaired goal-directed behaviour and habit formation in favour of the latter in clozapine-OCD as a potential theoretical framework for our results. Compulsion in clozapine-medicated schizophrenia patients could be understood as a long-term by-product of the psychosis (even after remission) perpetuated by clozapine’s potent antiserotoninergic properties. Screening for OCD in clozapine patients, and probably in those treated with structurally similar drugs like olanzapine, should be widely adopted by clinicians.

